# Building an inpatient addiction medicine consult service in Sudbury, Canada: preliminary data and lessons learned in the era of COVID-19

**DOI:** 10.1186/s13011-023-00537-y

**Published:** 2023-05-22

**Authors:** Tara Leary, Natalie Aubin, David C. Marsh, Michael Roach, Paola Nikodem, Joseph M. Caswell, Bridget Irwin, Emma Pillsworth, Maureen Mclelland, Brad Long, Sastry Bhagavatula, Joseph K Eibl, Kristen A. Morin

**Affiliations:** 1 Health Science North, Sudbury, Canada; 2 ICES North, Sudbury, Canada; 3 Health Sciences North Research Institute, Sudbury, Canada; 4 Northern Ontario School of Medicine, 936 Ramsey Lake Road, Sudbury, ON P3E 2C6 Canada

**Keywords:** Substance Use Disorder, Acute care, Addiction medicine consult service

## Abstract

**Objective:**

The goal of this study was to (1) Describe the patient population of a newly implemented addiction medicine consult service (AMCS); (2) Evaluate referrals to community-based addiction support services and acute health service use, over time; (3) Provide lessons learned.

**Methods:**

A retrospective observational analysis was conducted at Health Sciences North in Sudbury, Ontario, Canada, with a newly implemented AMCS from November 2018 and July 2021. Data were collected using the hospital’s electronic medical records. The outcomes measured included the number of emergency department visits, inpatient admissions, and re-visits over time. An interrupted time-series analysis was performed to measure the effect of AMCS implementation on acute health service use at Health Sciences North.

**Results:**

A total of 833 unique patients were assessed through the AMCS. A total of 1,294 referrals were made to community-based addiction support services, with the highest proportion of referrals between August and October 2020. The post-intervention trend for ED visits, ED re-visits, ED length of stay, inpatient visits, re-visits, and inpatient length of stay did not significantly differ from the pre-intervention period.

**Conclusion:**

Implementation of an AMCS provides a focused service for patients using with substance use disorders. The service resulted in a high referral rate to community-based addiction support services and limited changes in health service usage.

**Supplementary Information:**

The online version contains supplementary material available at 10.1186/s13011-023-00537-y.

## Introduction

Substance use is a significant public health concern in Northern Ontario, Canada, where there is limited access to health service providers [1–4]. Particularly, opioid use and related deaths are increasing exponentially [4–6]. In 2019, a Needs-Based Planning analysis for the Northeast Local Health Integration Network demonstrated that access to barrier-free health services for patients with substance use disorders (SUD) is lacking in Ontario, even more so in Northern Ontario [7]. Together with other reports and studies, the literature is clear that there is a need to expand health services and access to people with SUD, facilitate treatment uptake and retention, and reduce the growing rates of opioid-related adverse events [2, 3, 8, 9].

The greater city of Sudbury, the most populated city in Northern Ontario [10], has the highest rates of opioid-related emergency department (ED) visits and deaths are staggering and well above provincial averages [11]. This number has been steadily increasing every year. In 2019, the opioid overdose death rate was 28 per 100,000 people. In 2020 this number increased to 52.4 per 100,000 people. This increase has put pressure on the acute health care services in the area, as the number of individuals arriving at the ED and being admitted to the hospital has dramatically increased [11].

Inpatient Addiction Medicine Consult Services (AMCS) have been implemented across North America as one key strategy to help meet the needs of patients with SUDs in hospitals [2–13]. Like other subspecialty consultation services, the AMCSs collect data thorough histories, and examinations directly with patients or from treating physicians to provide evidence-based clinical recommendations for managing the presenting patient’s substance use disorder in hospital. Existing literature demonstrates that individuals with SUDs who receive a consultation from an inpatient addiction medicine consult service (AMCS)-like programs (compared to those who do not), demonstrate better engagement with primary care and HIV treatment following discharge, as well as reduced homelessness and increased number of days abstinent from drugs after discharge [12–15]. These services have also been shown to increase treatment uptake and post-discharge follow-up [3, 11], decrease disease severity [10], and potentially decrease re-admission rates and hospital costs [4, 8, 14].

Engaging SUD patients in acute care settings once medically stabilized has been shown to lead to decreased ED use and better transitions to outpatient treatment [16–19]. However, despite the rising incidence of SUD in hospitals and the benefits of engaging with patients during a hospital visit, problems with SUD are not often addressed [20–23]. While outpatient programs like rapid access to addiction medicine (RAAM) clinics [24, 25], addiction counselling, and opioid agonist treatment may be available in the community, physician or even self-referrals to such community programs remain uncommon [22, 23]. For instance, in a cross-sectional survey of internists, almost half reported caring for patients with SUD, and only 16% reported referring patients to treatment [26]. Without the support in hospital, patients are left to navigate their care after being discharged from the hospital, possibly hindering longer-term treatment uptake, leading to the well-documented high hospital and ED re-admission rates [11, 27–29].

Health Sciences North (HSN) in Sudbury, Ontario, Canada, implemented an AMCS in November 2019, a few months before the declaration of the global COVID-19 pandemic. Accordingly, this paper seeks to provide insight and guidance to others interested in establishing a similar model of care in their setting. More specifically, we aimed to (1) Describe the patient population that has benefitted from AMCS; (2) Evaluate ED use, inpatient admissions, length of stay, and referrals to community-based addiction supports over time; (3) Provide some lessons learned.

## Methods

### Design and setting

A retrospective observational analysis of the AMCS at HSN was conducted between November 2018 and September 2021. All adult patients over 18 years of age who presented to HSN and were referred to the AMCS during the study period were included in the analysis. Data were collected using the hospital’s electronic medical record (EMR). HSN is an acute care hospital located in Sudbury, Canada, which is considered a small urban setting in Northern Ontario. HSN is an academic health science center that services the catchment area for approximately 570,000 people across Northeastern Ontario. Ontario is Canada’s largest and most populated province, with over 85% of residents living in southern urban areas.

### The current model of care and program

The AMCS was initiated in November 2019, reaching full operation by January 2020. The service operates Monday to Friday, 8 am to 4 pm. The interdisciplinary team is comprised of consultant addiction physician specialists, a social worker, a nurse, and clerical support. The clinical backgrounds of each staff physician are varied and include psychiatry and family medicine. Additionally, the role of clinicians is embedded within the service to assist with motivational interviewing and psychosocial interventions related to a patient’s SUD (e.g. counselling, residential treatment, coverage for medication). One full-time addiction assessment nurse has been hired to support the team, to complete an assessment of any patient who has a SUD. The goal of the team is to address substance use issues and withdrawal-related symptoms as rapidly as possible to prevent patients from leaving the hospital prematurely. The clinical staff also work together to provide general addiction-related education, including trauma-informed training, harm-reduction and overdose prevention education (including the provision of a take-home naloxone kit), and education regarding community services to patients and staff with a longer-term goal of reducing stigma and improving access to care for all persons with SUD. Inpatient departments and the ED can consult the service electronically via an order entry through the hospital’s EMR system. At present, the service does not admit its patients and acts purely in consultation and follow-up capacity. The next development step is to embed after-hours and weekend services, but these were not implemented before the data collection period. Upon discharge from the hospital, patients are referred to community-based services including the rapid access addiction medicine service (RAAM), psychiatric services, referral to addiction treatment (opioid agonist treatment clinics or treatment programs), social supports (e.g., housing, social services), safe consumption supplies, naloxone kits, and other supportive support measures. The main supports available to the patient are summarized in Appendix A.

### Outcomes evaluation

Acute health service events were analyzed as outcomes using data routinely collected by HSN for the Canadian Institute for Health Information (CIHI)’s National Ambulatory Reporting System (NACRS) and Discharge Abstract Database (DAD). All hospital metrics were observed within the observation window only. ED usage metrics including monthly counts of ED visits, 30-day re-visits, the average length of stay in the ED, and the number of events deemed as left against medical advice (AMA), as well as inpatient metrics including monthly counts of hospital admissions, 30-day re-admissions to hospital, and the average length of stay in hospital were collected for all patients who presented to HSN and were referred to the AMCS between November 2019 and July 2021. NACRS and DAD codes are listed in Appendix B.

### Measurements

Outcomes were measured between November 2018 (one year before the implementation of AMCSs) and September 2021 (most recently available data). Descriptive statistics, including frequencies and proportions for categorical variables, and means and medians for continuous variables were calculated. Patient characteristics such as age, sex, overdose, and acute health service use were presented for all AMCS patients. Referrals to community programs.

Referrals from AMCS were counted monthly to provide context on the potential for the AMCS to provide specialized knowledge on how to support individuals after they leave the hospital. Referrals to the Rapid Access Addiction Medicine (RAAM) clinic, primary care providers, community addiction treatment, residential treatment, withdrawal management services, local opioid agonist treatment clinics, specialist care, internal HSN community counselling and mental health supports, community counselling and mental health supports external to HSN, social services, 12-step groups, private counselling, and crisis services were included in the study. Details on the services are provided in Appendix A.

### Statistical analysis

Data were aggregated into one-month periods for time series analyses. An interval of one month was chosen for creating the time series of acute health service use visits at HSN to ensure a reasonable number of events for adequate data points for quantitative analysis.

Interrupted time series (ITS) analyses were conducted for each outcome using a segmented regression approach [30–33]. The intervention for the current study was identified as the start of the AMCS program at HSN in November 2019. The data were split into pre-AMCS implementation and post-AMCS implementation periods. The pre-AMCS period corresponded to 12 months (November 2018 to October 2019), and the post-AMCS corresponded to 23 months (November 2019 to September 2021). Autocorrelation functions (up to lag 12) and Durbin-Watson tests were used to test for autocorrelation. Where autocorrelation was identified, ITS regression standard errors were adjusted using the Newey-West (1987) method. Model coefficients and their standard errors were used to calculate 95% confidence intervals (95% CI). All statistical analyses were performed using SAS on Demand for Academics, and results were considered statistically significant where p < 0.05. The study received approval through the Health Sciences North Research Institute Research Ethics Board.

## Results

### AMCS patient characteristics

A total of 833 unique patients were assessed through the AMCS. Of these patients, over half (533/833 = 63.99%) were male and the mean age was 42.6 years. The average monthly ED visits for overdoses in this cohort was 0.71 (1.77%), 3.20 (8.43%) for mental health-related visits, 1.41 (5.5%) for substance-related visits, and 7.85 (12.32%) times for ED visits other reasons. The average monthly inpatient hospital admissions for mental health was 1.84 (4.23%) for inpatient psychiatric admissions and 2.72 (3.97%) for inpatient admissions for other reasons. Results are presented in Table 1.

**Table 1:** Demographic information and health service use for adult patients over 18 years of age who presented to HSN and were referred to the AMCT between November 2019 and July 2021

Patient characteristics	
Sex *n (%)*	
*Male*	533 (63.99)
*Female*	300 (36.01)
Age *mean (STD)*	42.56 (14.99)
Average monthly overdose visits *mean (STD)*	0.71 (1.77)
Average monthly ED visits for mental health *mean (STD)*	3.20 (8.43)
Average monthly ED visits for substance use *mean (SD)*	1.41 (5.25)
Average monthly ED visits for all other reasons	7.85 (12.32)
Average monthly acute inpatient admission for mental health *mean (SD)*	1.84 (4.23)
Average monthly acute inpatient admission for all other reasons *mean (SD)*	2.72 (3.97)

*n = number

*STD = standard deviation

### Referrals to community-based addiction programs

**Fig. 1:**
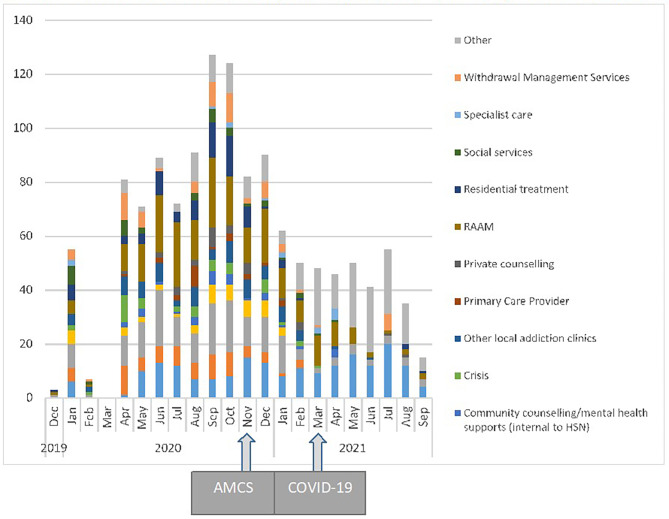
Number of patients referred out to community addiction programs from the Addiction Medicine Consultation Service (AMCS) at HSN from November 2018 to July 2021 (Sudbury, Canada)

Referrals were only counted after the implementation of AMCS (November 2019 to September 2021). During this observation period, there were 1,294 referrals made to community addiction support. Overall, referrals to community addiction support varied throughout the study period, with the highest proportion of referrals occurring between August and October 2020. The majority of AMCS referrals were to RAAM 17.0%) and again took place between June and December 2020 before decreasing after December 2020, followed by referrals to ‘other’ services (15.8%), and no referrals (15.1%). Detailed numbers are presented in Appendix C and illustrated graphically in Fig. 1.

### Emergency department usage

**Fig. 2:**
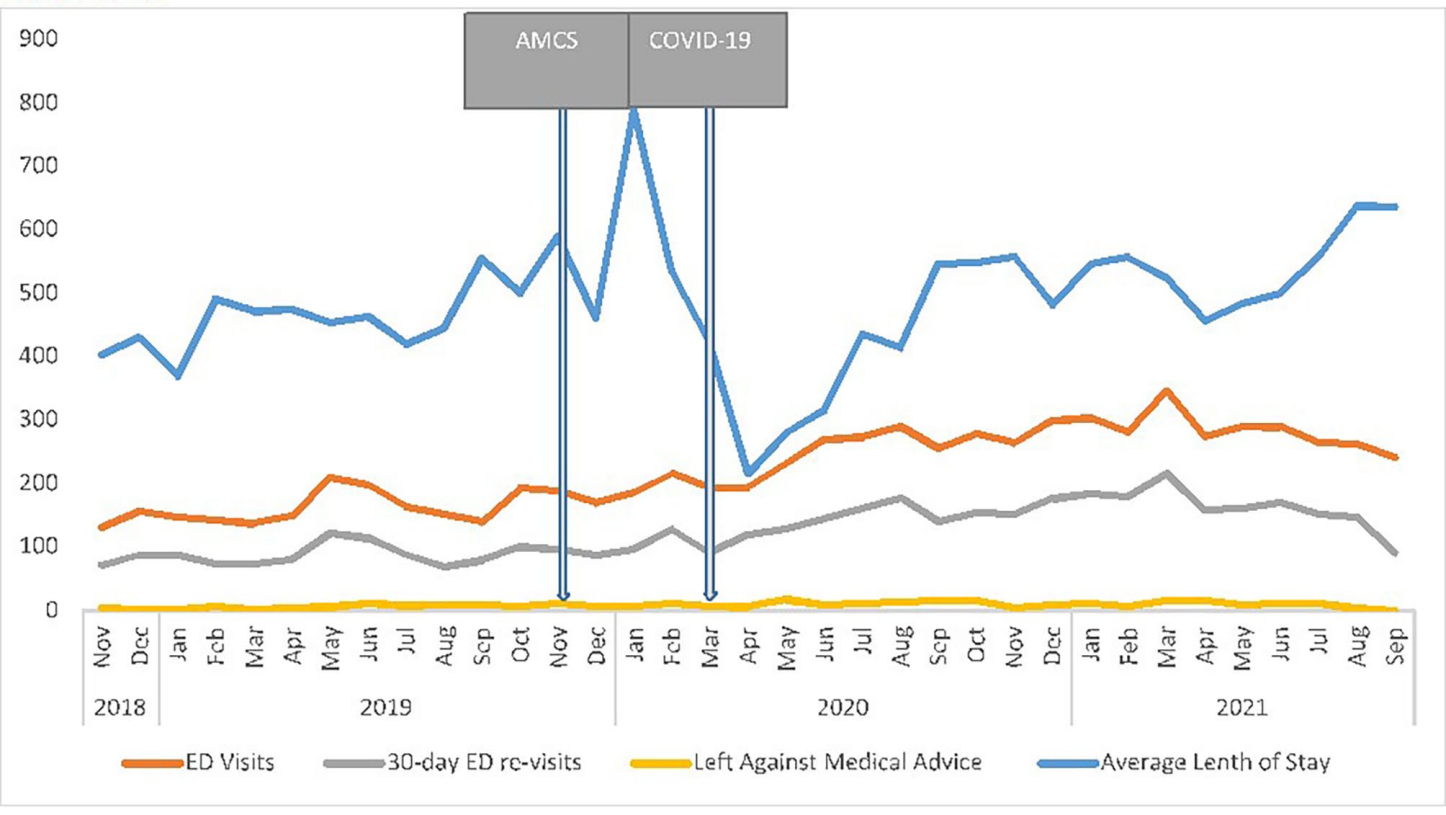
Number of ED visits, length of stay in ED, number of 30-day re-admissions ED at Health Sciences North from November 2018 to July 2021 (Sudbury, Canada); vertical centerline indicates the period of interruption (implementation of AMCS)

ED visits, re-visits, length of stay, and AMA events were plotted for AMCS patients before and after implementing the AMCS program (Fig. 2). The detailed results are listed in Table 2.

Single series ITS analysis of ED visits indicated no statistically significant trend before the implementation of AMCS (3.23, 95%CI −1.79 to 0.22). However, a particularly strong level change was observed immediately following the implementations, whereby ED visits increased by 21.15 (16.13 to 62.78). This increase was sustained over time with a moderate but insignificant post-interruption trend change of 1.42 (95% CI −3.59 to 6.79). On the other hand, no statistically significant trends in ED re-visit rates were observed before implementing the AMCS 1.17 (1.17, 95% CI −3.18 to 5.52). There was no significant change (18.42, 95% CI −23.19 to 60.03) immediately afterward. Although numbers continued to rise slightly before the implementation, there was no significant increase as indicated by the lack of post-interruption trend change (1.55, 95% CI −3.10 to 6.20). A single-series ITS analysis of the length of stay in ED was also conducted. Again, no significant trend was noted in the pre-interruption phase (4.79, 95% CI −18.16 to 27.74), nor was any apparent level change after the interruption period (−9.70, 95% CI −185.47 to 166.07). No significant post-interruption trend change of −0.78 (−27.32 to 25.76) was demonstrated. Lastly, a single-series ITS analysis of the number of AMA events was conducted. No significant trend was noted in the pre-interruption phase (1.05, 95% CI −3.79 to 5.89). However, a slight increase in the post-interruption level change was observed immediately following the implementation, increasing by 0.69 (0.02 to 1.36). A slight but statistically significant decrease in the number of AMA events of −0.77 (−1.48 to −0.06) was also demonstrated in the post-interruption trend change. All ITS model coefficients, along with their 95% CIs and the post-interruption trends, are presented in Table 2.

**Table 2:** Single ITS analysis for ED visits, re-visits, length of stay, and left against medical advice for AMCS patients

Emergency department			
	Visits β(95% CI)	Re-visits β(95% CI)	Length of stay β(95% CI)
β1 (pre-interruption trend)	3.23 (−1.79 to 0.22)	1.17 (−3.18 to 5.52)	4.79 (−18.16 to 27.74)
β2 (post-interruption level change	21.15 (16.13 to 62.78)	18.42 (−23.19 to 60.03)	*9.7 (185.47 to 166.07)
β3 (Post-interruption trend change)	1.42 (−3.59 to 6.79)	1.55 (−3.10 to 6.20)	*0.78 (−27.32 to 25.76
β1 + β3 (post-interruption trend)	4.65 (−0.36 to 16.99)	2.72 (−7.94 to 13.38)	4.31 (−2.28 to 10.90)

### Inpatient admissions

**Fig. 3:**
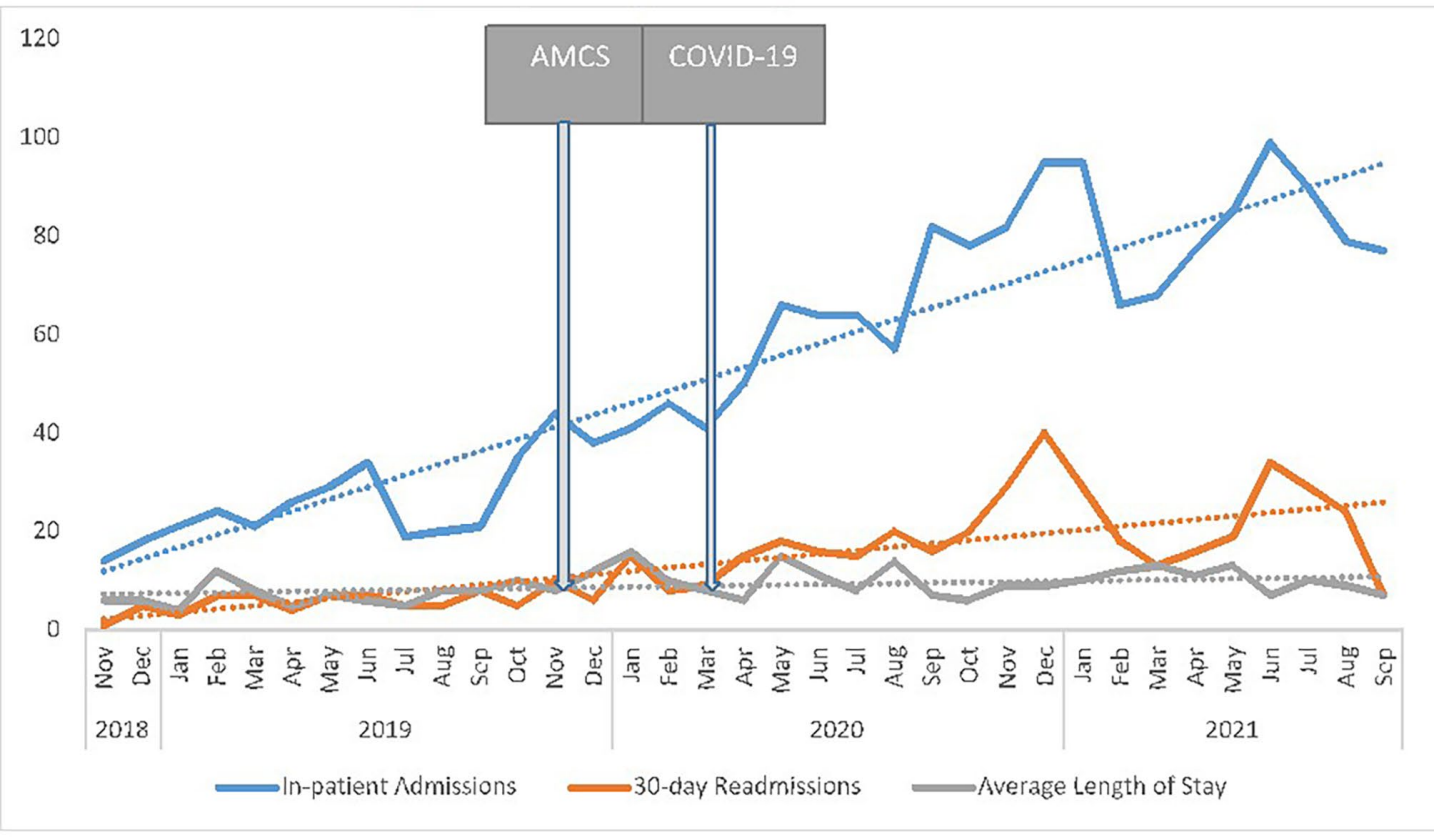
Number of inpatient admissions, re-admissions, and length of stay at Health Sciences North from November 2018 to July 2021 (Sudbury, Canada)

The number of hospital admissions, re-admissions, and length of stay for AMCS patients were assessed over time. Rates of ED use over time are plotted in Fig. 3. The detailed results are listed in Table 3.

ITS analysis of hospital admissions indicated no statistically significant trend before the implementation of the AMCS (0.94, 95% CI −0.67 to 2.08). A non-significant level change was observed immediately following the implementation, increasing by 13.00 (95% CI −0.31 to 8.17). This increase in inpatient hospitalizations was sustained over time, with a moderate but insignificant post-interruption trend change of 1.33 (−0.59 to 2.41). Rates of hospital re-admissions did not have any statistically significant trend before the AMCS was implemented (0.27, 95% CI −0.02 to 0.52), with no significant change (5.10, 95% CI −0.17 to 10.37) observed immediately afterward. There was no significant increase as indicated by the lack of post-interruption trend change (0.33, 95% CI −0.28 to 0.94). Single-series ITS analysis of the length of stay in the hospital was also conducted. Again, no significant trend was noted in the pre-interruption phase (0.18, 95% CI −0.27 to 1.02). As expected, there was no apparent level change after the interruption period (−2.71, 95% CI −0.97 to 3.02). The length of stay numbers also demonstrated a non-significant post-interruption trend change of −0.25 (−0.72 to 0.40). All ITS model coefficients and their 95% CIs and the post-interruption trends are presented in Table 3; Fig. 3.

**Table 3:** Single ITS analysis for hospital admissions, re-admissions, and length of stay for AMCT patients

In-patient Hospitalizations			
	Admissions β(95% CI)	Re-admissions β(95% CI)	Length of stay β(95% CI)
β1 (pre-interruption trend)	0.94 (−0.67 to 2.08)	0.27 (−0.02 to 0.52)	0.18 (−0.27 to 1.02)
β2 (post-interruption level change	13.00 (−0.31 to 8.17)	5.10 (−0.17 to 10.37)	2.71 (−0.97 to 3.02)
β3 (Post-interruption trend change)	1.33 (−0.59 to 2.41)	0.33 (−0.28 to 0.94)	*0.25 (−0.72 to 0.40)
β1 + β3 (post-interruption trend)	2.27 (−3.38 to 1.34)	0.60 (−1.71 to 2.91)	*0.07 (−1.43 to -0.07)

## Discussion

This study sought to describe the patient population that has benefitted from AMCS at HSN in Sudbury, Ontario, Canada; to highlight trends in referrals to community-based addiction support services, ED use, and inpatient use before and after the implementation of AMCS; as well as provide some lessons learned from implementing such services in the era of COVID-19.

Drawing on longitudinal data from HSN’s EMR, we identified distinct trajectories of referrals to addiction services, with all referrals peaking between August and October 2020. The results also demonstrated no statistically significant effect on ED and inpatient health care trends after implementing AMCS.

Although AMCS remains a developing form of hospital service, several major hospitals have implemented such services in their centers in recent years in response to the growing number of patients with SUD presenting to acute care settings [13]. To the best of our knowledge, HSN is the first Northern regional referral hospital in Ontario that serves rural areas to implement an AMCS. St. Paul’s Hospital in Vancouver has one of the largest and most long-standing AMCS in North America and reported serving a similar patient population, approximately 60% males in similar age categories [13].

A descriptive analysis identified that referrals to outpatient addiction support from AMCS peaked between August and October 2020. The trend observed in this analysis aligns with the timing of the AMCS implementation. The initiative started at HSN prior to the COVID-19 pandemic, with regional COVID-19 pandemic guidelines beginning March 15, 2020. Figure 1 demonstrated that referrals decreased dramatically between March and May 2020, which aligned with public health stay-at-home orders related to COVID-19. COVID-19 disproportionately burdens people with SUDs, making it more critical than ever to ensure patients are getting adequately treated and connected to care. Further consideration should be given to ways AMCS programs can adapt during the COVID-19 pandemic or other public health emergencies [20]. For instance, in Vancouver, Canada, Harris et al. (2021) recently published an article highlighting the need for a system, treatment, harm reduction, and discharge planning adaptations during a pandemic [34]. This included having telephone-based hospital consultations, providing longer buprenorphine bridge prescriptions at discharge with telemedicine follow-up appointments for tapers or alternatives, and increasing discharge outreach for high-risk patients through designated staff and technology.

Our secondary analysis demonstrated no statistically significant effect on ED and inpatient health care use trends after implementing AMCS. These findings can be explained by several factors, including the consideration of societal norms which focus on punitive measures rather than a compassionate understanding of SUDs, the limited hours of operation of the AMCS, the impact of COVID-19, and lastly, the effect of the increase in the availability and toxicity of synthetic opioids and drug combinations in the drug supply during the study period. Previous studies by Morin et al. showed very high rates of mental health problems among people with OUD [35–37]. And that these patients benefitted from reduced morbidity and mortality if they received mental health care from a psychiatrist or family physician while receiving addiction treatment [35, 37]. One of the roles of AMCS is to facilitate such coordinated care by arranging psychiatric consultation during inpatient admission if appropriate. Therefore, there is a possibility that inpatient admissions may have also increased because the introduction of the AMCS provided a more patient-centered and caring experience for patients when they required admission. This may mean that patients with SUDs would be more willing to return to hospital as some of the systemic barriers to accessing acute care when it is required, were reduced by the intervention of the AMCS. Further research is needed to understand patients’ perspectives on how hospital services help connect to community programs.

The evidence is clear that during our study period, the era of COVID-19, grief, isolation, income loss, and anxiety around the unknown triggered or exacerbated mental health conditions and led to increased levels of alcohol and drug use, insomnia, and anxiety [5, 38, 39]. Additionally, with the reduction of community-based support for people with mental health and SUDs, hospitals, and EDs became the main point of contact [40]. This combined with the reduction of support in the community for mental health and addictions led to devastating consequences such as increases in drug overdose deaths [11, 41]. The implementation of AMCS at HSN in Sudbury may have mitigated some of the high rates of acute care use during this time. However, more research is needed to support this assumption because HSN ED visit rates are similar to all of Ontario during the same time period. ED visits in Ontario increased by approximately 5.1% and at HSN by 4.6% from November 2018 to September 2021 [6].

It is essential to consider that hospital policy and importantly, clinician bias, ideologies, attitudes, and core beliefs are essential components to developing a successful AMCS [2, 5, 7, 15–17]. Due to legacy hospital policies on substance use, clinicians treating patients with SUD can be at odds with their patients’ needs. Considering there is a movement towards hospitals adopting socially accountable care, meaning that service provision addresses the priority health concerns of the population served [42], there is a discordance between the societal norms which focus on punitive measures for substance use and the needs of patients who have a physiologic dependence on a given substance (e.g., opioids). Thus, the culture shift within the hospital may be a significant, yet underappreciated, barrier to successfully implementing an AMCS. Therefore, longer-term mixed-methods evaluation is needed to understand culture change and staff buy-in and to evaluate outcomes over a longer period of time.

Currently, the AMCS only operates during weekdays, limiting the capacity to provide the service to those presenting after hours or on weekends. Priest and McCarty (2019) surveyed nine American hospitals with inpatient addiction medicine services. They found that only one service provided weekend consults, and most services did not provide coverage in the ED [7]. This is one area that may need to be further explored and developed as the program matures.

Finally, this implementation study took place at a single academic medical center in Northern Ontario, limiting its generalizability. However, to date, most implementation and evaluation studies have taken place at larger academic centres serving a predominantly urban population. HSN is a mid-sized academic centre that serves a large rural catchment area. This study may offer useful information for other mid-sized centres considering initiating an inpatient addictions medicine consult service. The AMCS model was implemented as a regional model at three other hospitals in Northeastern Ontario. Future studies should seek to include these other hospital programs.

### Limitations

Some limitations related to the intervention, data, and design merit discussion. Firstly, although AMCS is a solution to address the intensive needs of patients with a SUD, inpatient services alone are not sufficient to describe the impact of AMCS in its entirety, and having linkage to outpatient services was not available for this analysis. Linkage to outpatient data is a critical component to evaluate in future studies. Second, there are limitations associated with using administrative data to capture the effects of programs because of the potential for documenting and coding errors and oversight. Results could be supported in future studies with patient interviews or chart reviews. Third, ITS is a long-term analytical framework to examine data trends over time, better than a pre/post analysis. However, ITS cannot infer the causal effects of intervention especially if for events that coincide, and it can only make inferences at a population level.

### Lessons learned

The AMCS at HSN in Sudbury, Canada, was implemented during a time of collective trauma due to COVID-19, high overdose death rates, and an era of significant changes in health service delivery [5, 11, 43]. Accordingly, several important lessons have been learned.

The most notable lesson learned was that buy-in from the leadership team and their commitment to being socially accountable to the needs of their community was an essential component of this work. AMCS was implemented as a part of HSN’s strategic plan for 2019–2024. The strategic plan was developed based on consultation with 3,100 patients, employees, medical staff, learners, volunteers, foundations, and community partners. Despite minimal funding, it was prioritized in the organization in part due to buy-in from senior leadership and their commitment to being socially accountable to community needs.

Institutional (management and staff-level) buy-in through initiatives such as staff co-design, ongoing education initiatives, and the development of new policies to get was just as important as leadership buy-in to the successful implementation of the AMCS. The use of an interprofessional team to implement the AMCS strengthened the opportunities for patient-centered care. Staff, patient, and family input collected through surveys was also important in shaping shape the service. Engagement with staff also revealed an appetite by clinicians to improve how they care for people with SUD. Additionally, initiating treatment in the form of medications (e.g., methadone, buprenorphine/naloxone) to interested patients was feasible in the inpatient setting. The need for harm reduction and advocating for harm reduction was also highlighted, particularly for those patients who were not ready to engage in treatment discussions. This suggests that building organizational support and developing a supportive harm reduction policy are essential for other organizations. The need for mutual respect between patients and clinicians has also been a key factor in the implementation of AMCS. Commitment to these tenets was crystalized in a Harm Reduction Position Statement [44], which was adopted by hospital administration and staff in January of 2021 and ongoing education was provided throughout the organization surrounding this.

## Conclusion

In the wake of North America’s opioid epidemic, the COVID-19 pandemic, and its frequent intersection with acute care settings, hospitals offer a tremendous opportunity to initiate evidence-based care to manage not only opioid addiction but all SUD. AMCS provided an opportunity to improve the quality of care for patients with SUDs at HSN in Sudbury, Canada during a time of unprecedented population health emergencies due to COVID-19 and raising opioid overdoses. The AMCS was introduced at a time when the pandemic hit and substance use problems were exacerbated in the community due to stress, isolation, and closure of community support. Although no statistically significant changes in acute care use and referral patterns were observed, we believe that an inpatient AMCS at HSN provided a focused service for patients and mitigated potential upsurges in acute care re-visits. Additional research is needed on inpatient supports for patients with SUD and more information is needed to track patients after leaving the hospital and how the community supports impact hospital metrics and patient outcomes. This paper may offer insight and guidance to other health service providers interested in implementing a model of care in their setting to improve access to care for individuals with SUDs.

## Electronic supplementary material

Below is the link to the electronic supplementary material.

Supplementary Material 1

Supplementary Material 2

Supplementary Material 3

## Data Availability

The datasets used and/or analyzed during the current study are available from the corresponding author upon reasonable request.

## List of Abbreviations

AMA: Against medical advice
AMCS: Addiction medicine consult service
CI: Confidence Interval
CIHI: Canadian Institute for Health Information
DAD: Discharge Abstract Database
ED: Emergency department
EMR: Electronic medical record
HSN: Health Sciences North
ITS: Interrupted time series
NACRS: National Ambulatory Reporting System
PHSD: Public Health Sudbury & District’s
RAAM: Rapid access to addiction medicine
SUD: Substance use disorders
