# Supplementary material for: Building an inpatient addiction medicine consult service in Sudbury, Canada: preliminary data and lessons learned in the era of COVID-19
Source: Subst Abuse Treat Prev Policy. 2023 May 22;18:29. doi: 10.1186/s13011-023-00537-y (PMC10201028; doi:10.1186/s13011-023-00537-y)
Supplement: Supplementary file 2 — Supplementary Material 2 [file 13011_2023_537_MOESM2_ESM.docx]

Appendix A: Community-based addiction support services descriptions

| **Community-based addiction support services** | **Service Description** |
| --- | --- |
| RAAM | The Rapid Action to Addiction Medicine service provides quick access to care for substance use disorders, including assessments, counselling, prescriptions for medications that may help with decreasing cravings and withdrawal symptoms, and support through transitions. |
| Primary Care Provider | Includes referrals to all primary care practitioners, including physicians and nurse practitioners. |
| Community Addictions Treatment | Involves community-based services for substance use-specific counselling activities in individual or group formats. These sessions may be brief and sometimes offered on an outreach basis. They may be structured scheduled of substance use-specific counselling activities over some days/evenings, or partial days/evenings, of the week. |
| Residential Treatment | These services provide accommodations and engage clients in structured, scheduled interventions and activities specifically designed to ameliorate substance use problems and/or moderate the severity of co-occurring disorders. |
| Withdrawal Management Services | This involves outpatient treatment services providing for safe withdrawal in an ambulatory setting. |
| Other Local Addiction Clinics | Includes referrals to any local addiction clinics, including clinics providing opioid agonist treatment. |
| Specialist Care | Includes referrals to any physician-based specialist care. |
| Internal Community Counselling/Mental Health Supports | This involves community-based services internal to Health Sciences North for mental health-specific counselling activities in individual or group formats. These sessions may be brief and sometimes offered on an outreach basis. They may be structured scheduled of counselling activities taking place over some days/evenings, or partial days/evenings, of the week. |
| External Community Counselling/Mental Health Supports | This involves community-based services external to Health Sciences North for mental health-specific counselling activities in individual or group formats. These sessions may be brief and sometimes offered on an outreach basis. They may be structured scheduled of counselling activities taking place over some days/evenings, or partial days/evenings, of the week. |
| Social Services | This involves community-based social services external to Health Sciences North. The services may include housing, justice, income, human rights supports and case management. The sessions may be brief and sometimes offered on an outreach basis. |
| 12 Step Group | Twelve-step programs are mutual aid organizations for recovery from substance addictions, behavioral addictions and compulsions. |
| Private Counselling | Includes referrals to any private counselling services. |
| Crisis | The Crisis Intervention Program provides twenty-four-hour assessment and brief intervention services to individuals living with mental illness and those experiencing distressing feelings and thoughts related to harm towards self or others. Team members will meet with individuals and families to address their immediate situation and plan an effective action to resolve the crisis. If longer-term counselling, treatment of other disorders or hospital admission seems warranted, referral to services is offered. |
| Other | Includes any referrals made to programs or services that do not fit any definitions listed above. |
| No Referrals | Captured if not referral was made. |
