# Supplementary material for: Building an inpatient addiction medicine consult service in Sudbury, Canada: preliminary data and lessons learned in the era of COVID-19
Source: Subst Abuse Treat Prev Policy. 2023 May 22;18:29. doi: 10.1186/s13011-023-00537-y (PMC10201028; doi:10.1186/s13011-023-00537-y)
Supplement: Supplementary file 3 — Supplementary Material 3 [file 13011_2023_537_MOESM3_ESM.docx]

Appendix B: Variable definitions

| **Table** | **Column/Field** | **Description/Logic** | **Additional info** |
| --- | --- | --- | --- |
| **Table1_demographics** | Sex | Gender |  |
|  | Age | Age at 1st registration (Only PTs >= 18 included |  |
|  | ED_Overdoses_Visits | [# of HSN ED Visits where the ED Presenting Chief Complaint = 752 ( Page 2)](https://caep.ca/wp-content/uploads/2016/03/nacrs_presenting_complaint_list_v2_0_en_fr_pdf_pdf.pdf) | https://caep.ca/wp-content/uploads/2016/03/nacrs_presenting_complaint_list_v2_0_en_fr_pdf_pdf.pdf |
|  | ED_Substance Abuse_Visits | [# of HSN ED Visits where the ED Presenting Chief Complaint in (751, 753) - Page 2](https://caep.ca/wp-content/uploads/2016/03/nacrs_presenting_complaint_list_v2_0_en_fr_pdf_pdf.pdf) | https://caep.ca/wp-content/uploads/2016/03/nacrs_presenting_complaint_list_v2_0_en_fr_pdf_pdf.pdf |
|  | ED_Mental Health_Visits | [# of HSN ED Visits where the ED Presenting Chief Complaint between 351 and 400 - Page 1](https://caep.ca/wp-content/uploads/2016/03/nacrs_presenting_complaint_list_v2_0_en_fr_pdf_pdf.pdf) | https://caep.ca/wp-content/uploads/2016/03/nacrs_presenting_complaint_list_v2_0_en_fr_pdf_pdf.pdf |
|  | Other_ED_Visits | # of HSN ED Visits where the ED Presenting Chief Complaint not between 351 and 400, 351-353 |  |
|  | Mental_Health_Inpt_Admissions | [# of MH Inpt admissions to the MH&A Institution 4560 (All HSN MH Adults admissions have an account that startswith "SA".](https://www.health.gov.on.ca/en/common/ministry/publications/reports/master_numsys/mnb.xlsx) | https://www.health.gov.on.ca/en/common/ministry/publications/reports/master_numsys/mnb.xlsx |
|  | Acute_Inpt_Admission | [# of Inpt admissions to the Acute Institution 4059 (All HSN MH Adults admissions have an account that startswith "SA".](https://www.health.gov.on.ca/en/common/ministry/publications/reports/master_numsys/mnb.xlsx) | https://www.health.gov.on.ca/en/common/ministry/publications/reports/master_numsys/mnb.xlsx |
| **Table2_Referral Consults** | 7 - No Referrals | # of records where No Order Entries requests were found |  |
|  | Columns C to Q | # of records - based on non the Order Entry entered (see Appendix A |  |
| **Table3_ED_Results** | MonthPeriod | Month in Question |  |
|  | ED_Visits | # of Total ED visits in the month in question for the AMCT patient population |  |
|  | ED_LAMA | [# of ED Visits with DischargeDisposition in ('05','62','64') - Page 9= 05, Page 10 = 62,64](https://www.cihi.ca/sites/default/files/document/cihi-portal-release-notes-release-13.1-en_en02jpdg.pdf) | https://www.cihi.ca/sites/default/files/document/cihi-portal-release-notes-release-13.1-en_en02jpdg.pdf |
|  | ED_30day_Revisit | # of ED discharges where a subsequent ED registration occurred within 30 days. No filters on diagnosis |  |
|  | AVG_ED_LOS_Minutes | Avg # of minutes of time spent in the ED (Registiration to Depart/decision to admit) |  |
| **Table4_Acute_Results** | Month/Period | Month in Question |  |
|  | Acute_Admissions | # of admissions in the month for the AMCT patient population |  |
|  | Acute_AMA | DischargeDisposition in ('05','62','64') in the DAD abstract |  |
|  | 30_Day_Readmissions | # of admissions that occurred that month where when the pt was discharged, he was re-admitted (Acute) within 30 days of the discharge |  |
|  | AVG_ACUTE_LOS_Days | Avg # of days spent admitted as an Acute inpatient |  |
